# Ovarian tumor diagnosis using deep convolutional neural networks and a denoising convolutional autoencoder

**DOI:** 10.1038/s41598-022-20653-2

**Published:** 2022-10-11

**Authors:** Yuyeon Jung, Taewan Kim, Mi-Ryung Han, Sejin Kim, Geunyoung Kim, Seungchul Lee, Youn Jin Choi

**Affiliations:** 1 Department of Obstetrics and Gynecology, Soonchunhyang University Bucheon Hospital, Soonchunhyang University College of Medicine, Bucheon, Republic of Korea; 2 Department of Mechanical Engineering, Pohang University of Science and Technology (POSTECH), Pohang, Republic of Korea; 3 Division of Life Sciences, College of Life Sciences and Bioengineering, Incheon National University, Incheon, Republic of Korea; 4 Department of Obstetrics and Gynecology, Seoul St. Mary’s Hospital, College of Medicine, The Catholic University of Korea, Seoul, Republic of Korea; 5 Graduate School of Artificial Intelligence, Pohang University of Science and Technology (POSTECH), Pohang, Republic of Korea; 6 Institute of Convergence Research and Education in Advanced Technology, Yonsei University, Seoul, Republic of Korea; 7 Cancer Research Institute, College of Medicine, The Catholic University of Korea, Seoul, Republic of Korea

**Keywords:** Biological techniques, Computational biology and bioinformatics

## Abstract

Discrimination of ovarian tumors is necessary for proper treatment. In this study, we developed a convolutional neural network model with a convolutional autoencoder (CNN-CAE) to classify ovarian tumors. A total of 1613 ultrasound images of ovaries with known pathological diagnoses were pre-processed and augmented for deep learning analysis. We designed a CNN-CAE model that removes the unnecessary information (e.g., calipers and annotations) from ultrasound images and classifies ovaries into five classes. We used fivefold cross-validation to evaluate the performance of the CNN-CAE model in terms of accuracy, sensitivity, specificity, and the area under the receiver operating characteristic curve (AUC). Gradient-weighted class activation mapping (Grad-CAM) was applied to visualize and verify the CNN-CAE model results qualitatively. In classifying normal versus ovarian tumors, the CNN-CAE model showed 97.2% accuracy, 97.2% sensitivity, and 0.9936 AUC with DenseNet121 CNN architecture. In distinguishing malignant ovarian tumors, the CNN-CAE model showed 90.12% accuracy, 86.67% sensitivity, and 0.9406 AUC with DenseNet161 CNN architecture. Grad-CAM showed that the CNN-CAE model recognizes valid texture and morphology features from the ultrasound images and classifies ovarian tumors from these features. CNN-CAE is a feasible diagnostic tool that is capable of robustly classifying ovarian tumors by eliminating marks on ultrasound images. CNN-CAE demonstrates an important application value in clinical conditions.

## Introduction

Ovarian tumors affect women of all ages and pose a difficult diagnostic challenge for gynecologists. Once an ovarian mass is detected, the clinician’s priority is to determine whether it is a benign or a malignant tumor and to assess the options for optimal management^1^. Pelvic ultrasonography, which provides information about the tumor such as its size, consistency, and nature, is the most commonly used imaging approach in assessing ovarian tumors. The major limitation of ultrasonography is that it is operator-dependent and results can vary between interpreters.

Recent research has examined the value of texture analysis in the detection of cervical neoplasms on colposcopic images^2^ and the identification of breast lesions on digital X-ray mammograms through deep learning^3^. Khazendar et al.^4^ developed a support vector machine (SVM) to distinguish benign and malignant lesions by using 187 ultrasound images of ovarian tumors, and a three-dimensional texture analysis algorithm has been developed to evaluate structural changes in the extracellular matrix between normal ovary and serous ovarian cancer^5^.

Not only is it important to differentiate benign from malignant ovarian tumors, it is also important to distinguish among the various benign ovarian tumor types, because it is estimated that up to 10% of women will have surgery for an ovarian cyst in their lifetime^6^. Therefore, in this study, we aimed to accurately diagnose malignant and various benign ovarian tumors by using a texture analysis approach on ultrasound images^7^.

The most important thing in texture analysis is the quality of the image. Any disturbances on an image can interfere with texture feature learning, and it is inevitable that among the many images collected for large cohort studies^8^, there will be images with disturbances. These disturbances are not easily removed manually, as the phenotypes of disturbances are different from each other. This study proposes a deep learning-based approach, a convolutional neural network with a convolutional autoencoder (CNN-CAE), to remove disturbances automatically and sort ovarian tumors into five classes: normal (no lesion), cystadenoma, mature cystic teratoma, endometrioma, and malignant tumor.

The effectiveness of the proposed CNN-CAE is validated across 1613 ovarian ultrasound images collected from 1154 patients. The deep learning visualization method and degradation of sorting performance validate the effect of image disturbance on texture analysis qualitatively and quantitatively. We believe that the CNN-CAE we propose is a viable deep learning-based diagnostic tool for distinguishing ovarian tumors.

The remainder of this paper is organized as follows. Section “Results” presents the results of this study. Section “Discussion” presents the discussion. Section “Material and methods” describes the material and methods.

## Results

### Removing marks via CAE

**Figure 1:**
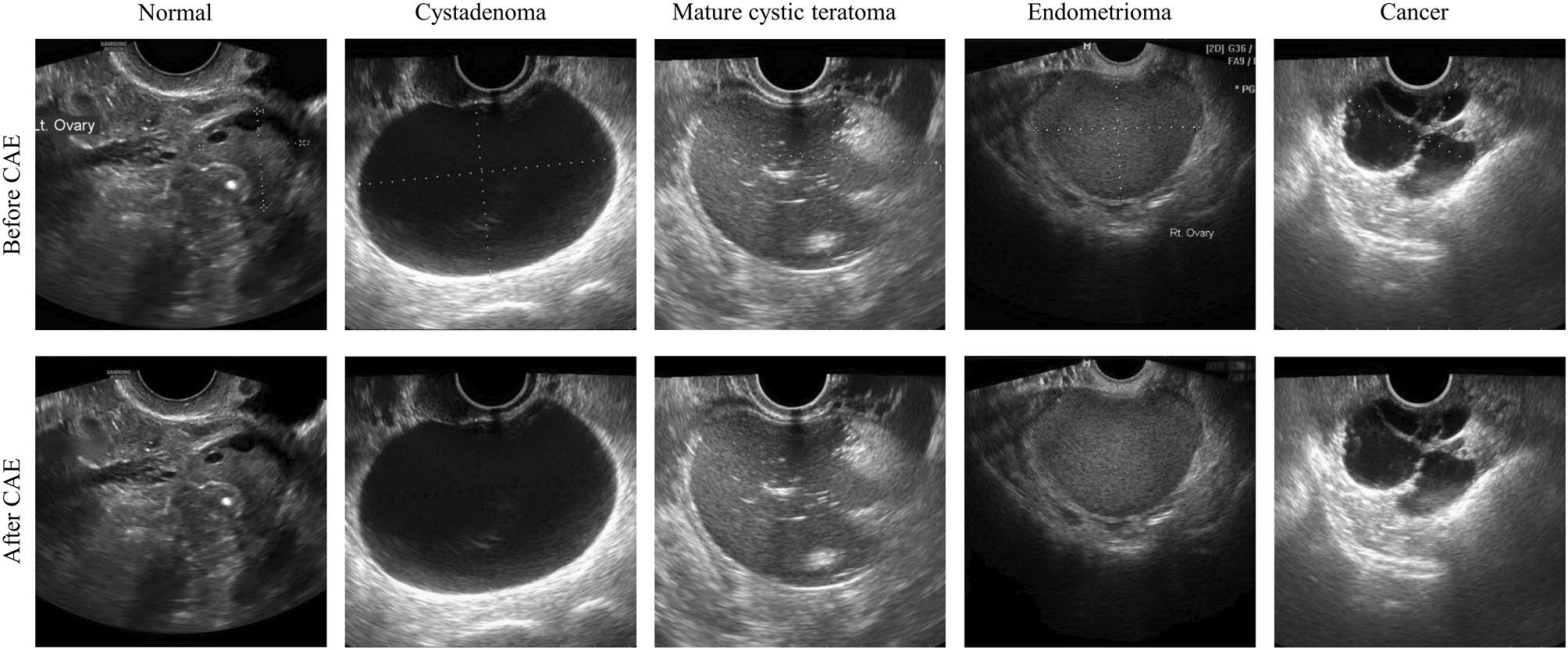
Ultrasound images before and after removing the marks via the convolutional autoencoder. The first row is the images with marks, and the second row is the image without marks. Example images are from left to right normal, cystadenoma, mature cystic teratoma, endometrioma, and malignancy.

Figure 1 shows the images before and after removal of marks via the CAE model. There are calipers and annotations around the ovary on the upper images, which inevitably affect the features that the CNN model learns. As seen in the lower row, the images we obtained through the CAE model are relatively clean, without calipers or annotations around the ovary. The pixels replacing the marks are well generated compared with the surrounding pixels, without a sense of heterogeneity.

### Classification results of CNN-CAE

The DenseNet classification results are shown in Table 1. In classifying normal versus other ovaries, the DenseNet121 model showed an accuracy of 97.22% with an area under the receiver operating characteristic curve (AUC) of 0.9936, sensitivity of 97.22%, and specificity of 97.21% the DenseNet161 model showed an accuracy of 97.28% with an AUC of 0.9918, sensitivity of 90.70%, and specificity of 98.29%. In determining tumor malignancy, the DenseNet121 model showed an accuracy of 90.49% with an AUC of 0.9395 and the DenseNet201 model showed an accuracy of 88.7% with an AUC of 0.9419. In the classification of the different types of benign tumors, the DenseNet161 model showed 82.18% sensitivity and an AUC of 0.9394 for cystadenoma, 80.82% sensitivity with an AUC of 0.9414 for mature cystic teratoma, and 73.33% sensitivity with an AUC of 0.9248 for endometrioma.

**Table 1:** Diagnostic performance and 95% confidence intervals (in parentheses) of the DenseNet models.

Outcome measure	Accuracy (%)	Sensitivity (%)	Specificity (%)	PPV (%)	NPV (%)	AUC
**Normal**						
DenseNet121	97.22 (96.68–97.76)	97.22 (96.85–97.59)	97.21 (94.79–99.63)	84.28 (82.37–86.19)	99.56 (99.19–99.94)	0.9936 (0.9914–0.9958)
DenseNet161	97.28 (96.37–98.2)	90.70 (86.13–95.26)	98.29 (97.64–98.95)	89.1 (85.24–92.95)	98.57 (97.88–99.27)	0.9918 (0.988–0.9956)
DenseNet201	96.98 (96.41–97.54)	89.3 (85.43–93.18)	98.15 (97.78–98.52)	88.1 (85.97–90.23)	98.36 (97.78–98.94)	0.9909 (0.9878–0.9940)
DenseNet161(no CAE)	95.25 ( 94.81–95.68)	89.3 (86.01–92.59)	96.16 (95.43–96.88)	78.14 (75.26–81.01)	98.33 (97.83–98.83)	0.9906 (0.9885–0.9927)
**Cystadenoma (benign)**						
DenseNet121	92.72 (91.92–93.51)	80.36 (77.42–83.31)	95.24 (94.64–95.84)	77.56 (75.14–79.98)	95.96 (95.37–96.55)	0.9213 (0.9062–0.9364)
DenseNet161	93.02 (92.59–93.46)	82.18 (77.03–87.33)	95.24 (93.96–96.52)	78.11 (74.56–81.67)	96.33 (95.33–97.33)	0.9394 (0.9272–0.9515)
DenseNet201	92.10 (91.35–92.85)	68.00 (64.22–71.78)	97.03 (96.16–97.89)	82.51 (78.36–86.66)	93.69 (93.0–94.37)	0.9423 (0.9324–0.9521)
DenseNet161(no CAE)	88.83 (88.0–89.66)	68.0 (63.05–72.95)	93.09 (92.56–93.61)	66.77 (64.77–68.78)	93.44 (92.5–94.37)	0.9109 (0.9076–0.9143)
**Mature cystic teratoma (benign)**						
DenseNet121	91.85 (91.21–92.49)	77.53 (76.6–78.47)	96.02 (95.23–96.8)	85.02 (82.51–87.53)	93.63 (93.38–93.88)	0.9409 (0.9390–0.9429)
DenseNet161	92.47 (91.77–93.17)	80.82 (77.88–83.77)	95.86 (95.11–96.61)	85.05 (82.84–87.26)	94.51 (93.73–95.28)	0.9326 (0.9247–0.9404)
DenseNet201	88.40 (87.0–89.79)	75.89 (70.84–80.94)	92.03 (91.11–92.96)	73.47 (70.82–76.13)	92.93 (91.55–94.31)	0.9144 (0.8951–0.9338)
DenseNet161(no CAE)	87.1 (85.49–88.71)	72.33 (68.64–76.02)	91.39 (90.31–92.48)	70.97 (67.4–74.54)	91.9 (90.84–92.97)	0.9056 (0.8972–0.914)
**Endometrioma (benign)**						
DenseNet121	93.64 (92.41–94.87)	79.56 (75.96–83.15)	95.91 (94.59–97.23)	76.08 (69.94–82.22)	96.68 (96.11–97.25)	0.9398 (0.9245–0.9550)
DenseNet161	93.64 (92.95––94.34)	73.33 (70.57–76.09)	96.92 (96.17–97.66)	79.43 (75.36–83.5)	95.75 (95.33–96.17)	0.9248 (0.9097–0.9400)
DenseNet201	92.84 (92.52–93.16)	76.89 (74.42–79.36)	95.41 (94.83–95.99)	73.04 (71.08–75.01)	96.24 (95.87–96.61)	0.9311 (0.9235–0.9388)
DenseNet161(no CAE)	91.79 (90.75–92.83)	76.8 (74.42–79.36)	94.19 (93.23–95.16)	68.19 (64.37–72.01)	96.19 (95.78–96.6)	0.9238 (0.9188–0.9288)
**Borderline/cancer (malignant)**						
DenseNet121	90.49 (88.9–92.08)	83.7 (78.72–88.69)	93.89 (92.94–94.83)	87.27 (85.67–88.87)	92.05 (89.8–94.29)	0.9395 (0.9293–0.9497)
DenseNet161	90.12 (88.39–91.86)	86.67 (81.0–92.33)	91.85 (91.2–92.51)	84.17 (83.15–85.18)	93.28 (90.55–96.01)	0.9406 (0.9269–0.9542)
DenseNet201	88.7 (87.47–89.93)	85.0 (81.92–88.08)	90.56 (89.9–91.21)	81.81 (80.54–83.09)	92.36 (90.88–93.84)	0.9419 (0.9356–0.9483)
DenseNet161(no CAE)	85.43 (84.75–86.12)	71.48 (69.59–73.37)	92.41 (91.89–92.92)	82.48 (81.46–83.51)	86.64 (85.87–87.4)	0.9093 (0.8995–0.9191)

*PPV* positive predictive value, *NPV* negative predictive value, *AUC* area under the receiver operating characteristic curve.

**Figure 2:**
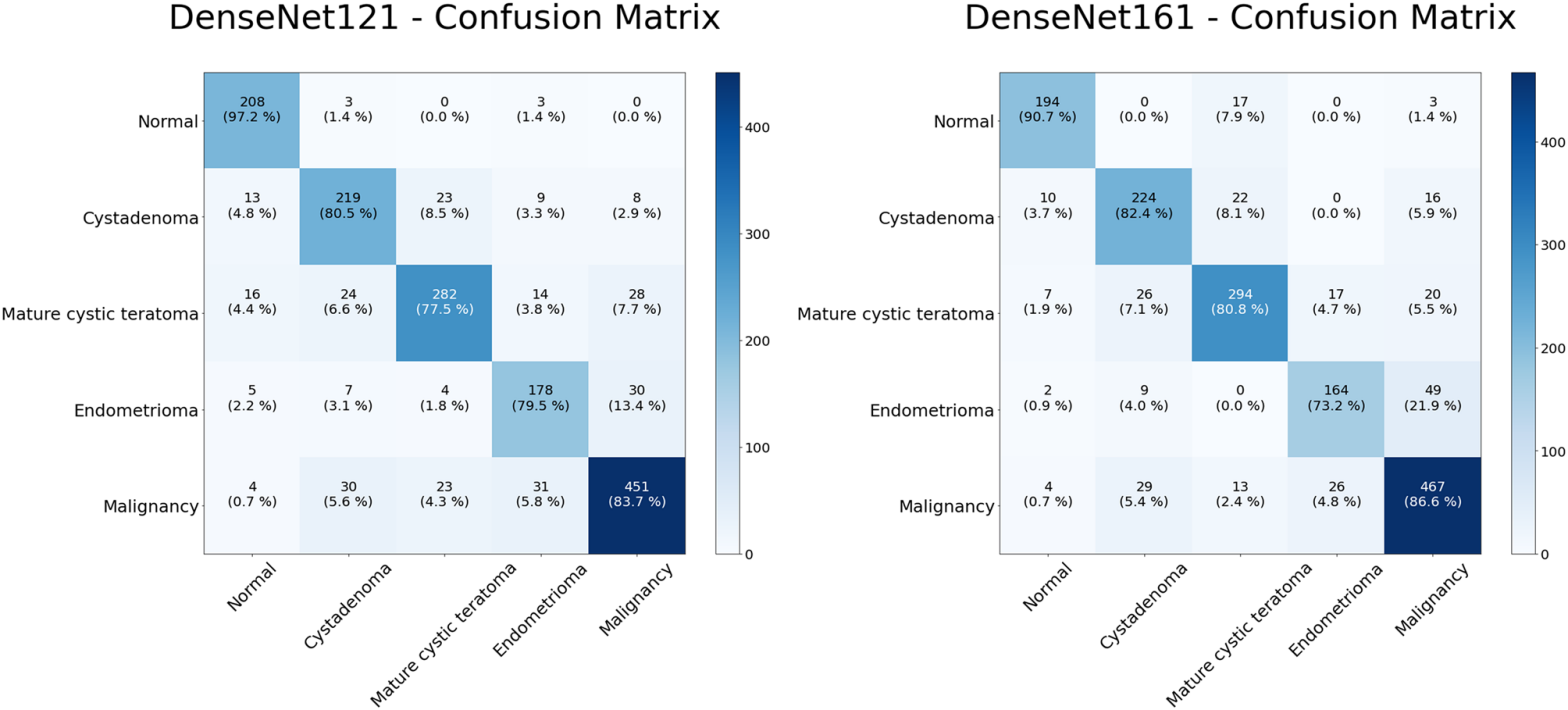
Multiclass classification of ultrasound images. Heat map of the confusion matrices of two highest performance models, DenseNet121 and DenseNet161.

The classification results for the two highest performance models are shown on the confusion matrices in Fig. 2. The predictions by the model are shown on the X-axis and the pathology diagnoses are shown on the Y-axis. As can be seen from these diagrams, the DenseNet161 model showed a better result in the classification of malignancies. Malignancies were correctly identified in 467 of 539 images (86.6%). The DenseNet121 model correctly classified 451 (83.7%) lesions as malignant. For benign tumors, approximately 70.0–80.0% of the images were correctly sorted into each class. In the sorting of benign tumors, the DenseNet161 model correctly classified 682 images out of 860 images, while the DenseNet121 model classified 679 images out of 860 images correctly.

**Figure 3:**
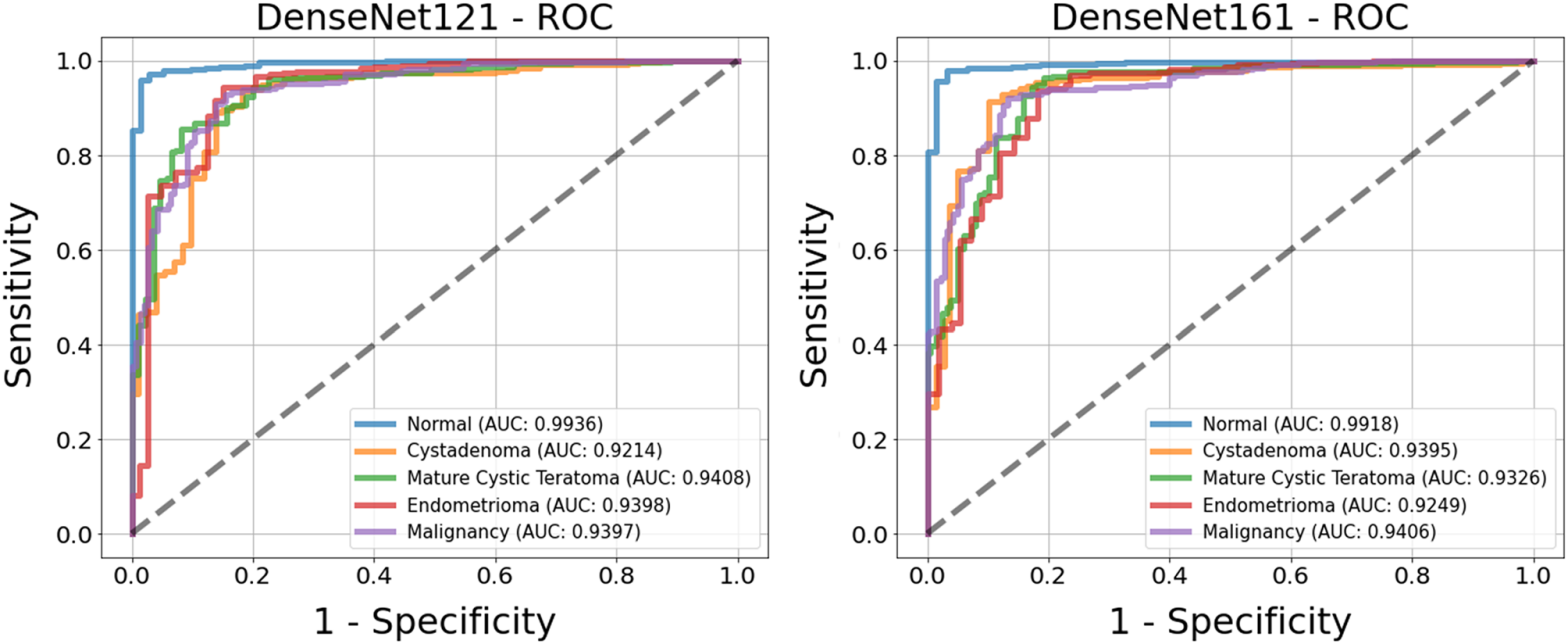
Receiver operating characteristic (ROC) curves of the classification results for two models, DenseNet121 (left) and DenseNet161 (right). The ROC curves are based on the binary results for each class.

Comparison of the receiver operating characteristic curves (ROC) for the two top models is shown in Fig. 3. The AUC values for each class of both models are in the range of 0.89–0.98. In determining tumor presence, the DenseNet161 model had an AUC of 0.9837, indicating that the presence of a tumor is well distinguished. The ROC for tumor malignancy had an AUC > 0.9, which is promising for distinguishing malignant tumors.

### Validation of CNN-CAE through the visualization method

**Figure 4:**
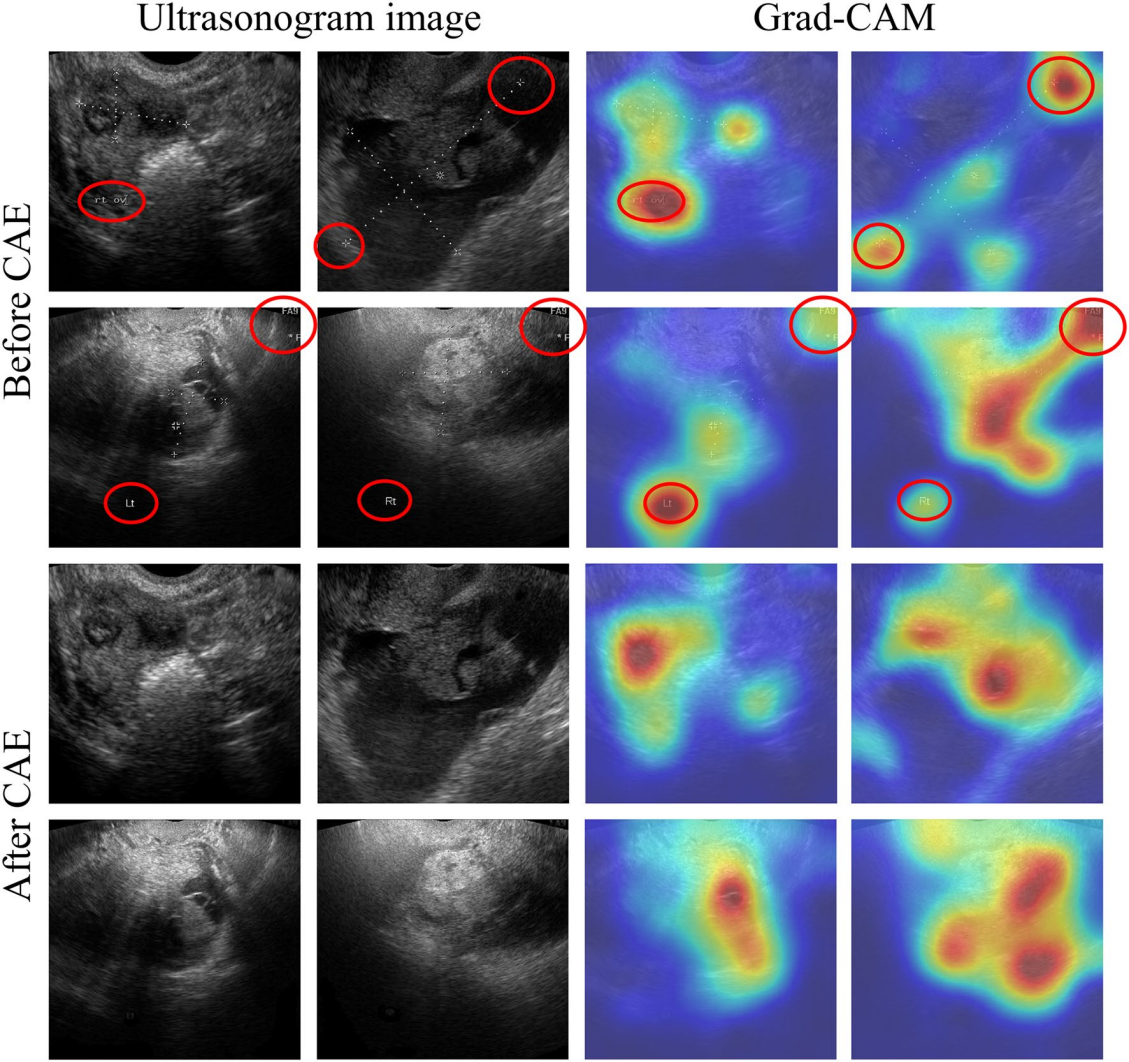
Convolutional neural network visualization of ultrasound images via a gradient-weighted class activation map (Grad-CAM). The first two rows (“Before CAE”) show the images with marks and the corresponding Grad-CAM results, and the next two rows (“After CAE”) show the images with marks removed and the corresponding Grad-CAM results.

We applied a gradient-weighted class activation mapping (Grad-CAM)^9^ CNN visualization method to determine the effects of marks on CNN learning. For comparison, we trained the same CNN model with the images without removing the marks and visualized both CNN models. The visualization results are shown in Fig. 4. The images on the left are input images, and those on the right are visualization results through Grad-CAM. In the Grad-CAM image, the activated (red) area is strongly considered in predicting final results, whereas the blue area is generally not considered in the final result. The Grad-CAM result shows that marks on the images, such as calipers and annotation, can affect the classification process in the CNN model. Furthermore, we could see that the activation area of the model trained without marks coincided with the correct area more often. This means that the classification is based on morphology and texture information, and thus we can regard the classification results are valid. However, the activation area of the model trained with marks is distributed over an incorrect area, as can be seen in Fig. 4 (red circles), which means that the classification is not based on shape and texture information. Therefore, these results are considered invalid.

### Validation of CNN-CAE through classification accuracy

For quantitative evaluation of the effect of the marks, we tested the classification performance the best-performing model, DenseNet161, without CAE. The results are shown in in Table 1. Compared to the DenseNet models with CAE, DenseNet161 without CAE showed lower accuracy (95.25%) for classifying normal versus other ovarian tumors and showed 71.48% sensitivity for detecting malignancy, with an AUC of 0.9093.

### Diagnostic accuracy based on the interpreter’s experience

All of the ultrasound images were independently interpreted by novice, intermediate, and advanced readers. The novice readers had two years of experience interpreting gynecologic ultrasound, the intermediate readers had 5 years of experience, and the advanced readers had ten years of experience. Two interpreters served as readers for each group. As shown in Table 2, the novice readers had an accuracy of 66.8%, the intermediate readers had an accuracy of 86.9%, and the advanced readers obtained an accuracy of 91.0%.

**Table 2:** Diagnostic performance of interpreters of ultrasonography.

	Accuracy (%)	Normal (%)	Cystadenoma (%)	Mature cystic teratoma (%)	Endometrioma (%)	Malignancy (%)
Beginner	66.8	96.3	54.1	59.2	56.6	67.9
Intermediate	86.9	99.6	56.1	88.7	94.8	95.3
Advanced	91.0	97.6	84.3	89.1	89.9	94.0

## Discussion

The correct diagnosis of ovarian tumors is necessary for determining the appropriate treatment, and several machine learning methods have been studied for ovarian tumor diagnosis. It is of utmost importance to distinguish malignancies among the various ovarian tumors. Previous machine learning trials have distinguished only between benign and malignant tumors in small populations. In two papers from the Timmerman group evaluating the differentiation of ovarian tumors using machine learning, the first study showed 76.0% accuracy using SVM on 187 ultrasound images^10^, and the second reported an accuracy of 77.0% using a local binary pattern coding operator^4^. However, the ultrasound images were based on the segmentation of lesions and displayed calipers used by the ultrasound specialists, which presents the limitation of an intervention bias. Another recent study reported that the manual removal of peripheral organs (e.g., uterus) from the image resulted in a sensitivity of 96.0% and a specificity of 89.3% for distinguishing between benign and malignant ovarian tumors^11^, but it is important not only to distinguish between malignant and benign tumors, but also to identify benign tumors that require surgery and those that may progress to malignancies. We performed deep learning analysis in one normal ovary and four common ovarian tumors, and our CNN-CAE study differentiated four types of ovarian tumor that required surgical treatment with an accuracy of 97.0%. In particular, we determined that the AUC for malignancy was 0.94, which clearly distinguished malignant from benign. Comparing the CNN-CAE results with the reading results of novice, intermediate, and advanced readers showed an accuracy of 66.8%, 86.9%, and 91.0%, respectively, suggesting that, considering the CNN-CAE results, inexperienced examiners can diagnose ovarian tumors with high accuracy.

Deep learning is vulnerable to imperceptible perturbations of input data. Some studies have shown that small disturbances to the input data can significantly degrade deep learning performance^12^. In this study, CNN-CAE was used to remove marks from ultrasound images to improve diagnostic accuracy. We confirmed that disturbances such as calipers and annotations could affect the CNN model results via Grad-CAM, and we developed the CNN-CAE model to eliminate these calipers and annotations. The CNN-CAE model successfully removed the calipers and annotations and classified ultrasound images at a high level of accuracy. These results show that even if marks are present on ultrasound images, they can be removed automatically so that only the ovary can be assessed for the correct diagnosis. The visualization results of Grad-CAM verify the reliability of the CNN-CAE model in terms of utilizing data in which disturbances exist. These results show that our method no longer needs to save a no-caliper image when taking an ultrasound image, and has the advantage of utilizing existing stored caliper images in addition to the images taken on-site. Our study is the first to address the removal of disturbances on ovarian ultrasound images via deep learning, and we have also demonstrated the usefulness of a deep learning-based method to solve the problem of disturbances existing in medical data.

There are some limitations to this study. First, this study was a retrospective cohort study performed on images of tumors with a known histological diagnosis, and was performed on a relatively small number of patients and images. Second, only still ultrasound images were used. Therefore, the multi-focal images that can be extracted from the ultrasound video were not fully utilized. Nevertheless, the CNN-CAE model has the potential to be widely used not only to identify malignancy but also for the classification of benign tumors that require surgery. Furthermore, the model has the advantage that it can be read not only in newly captured ultrasound images but also previously captured images. In future research, we will develop a classification model based on the most recent method and examine various aspects of ovarian tumor imaging, such as clinical radiology and ultrasound imaging technique. In addition, we will conduct a study to improve classification accuracy using multi-focal images.

## Material and methods

### Overall process

**Figure 5:**
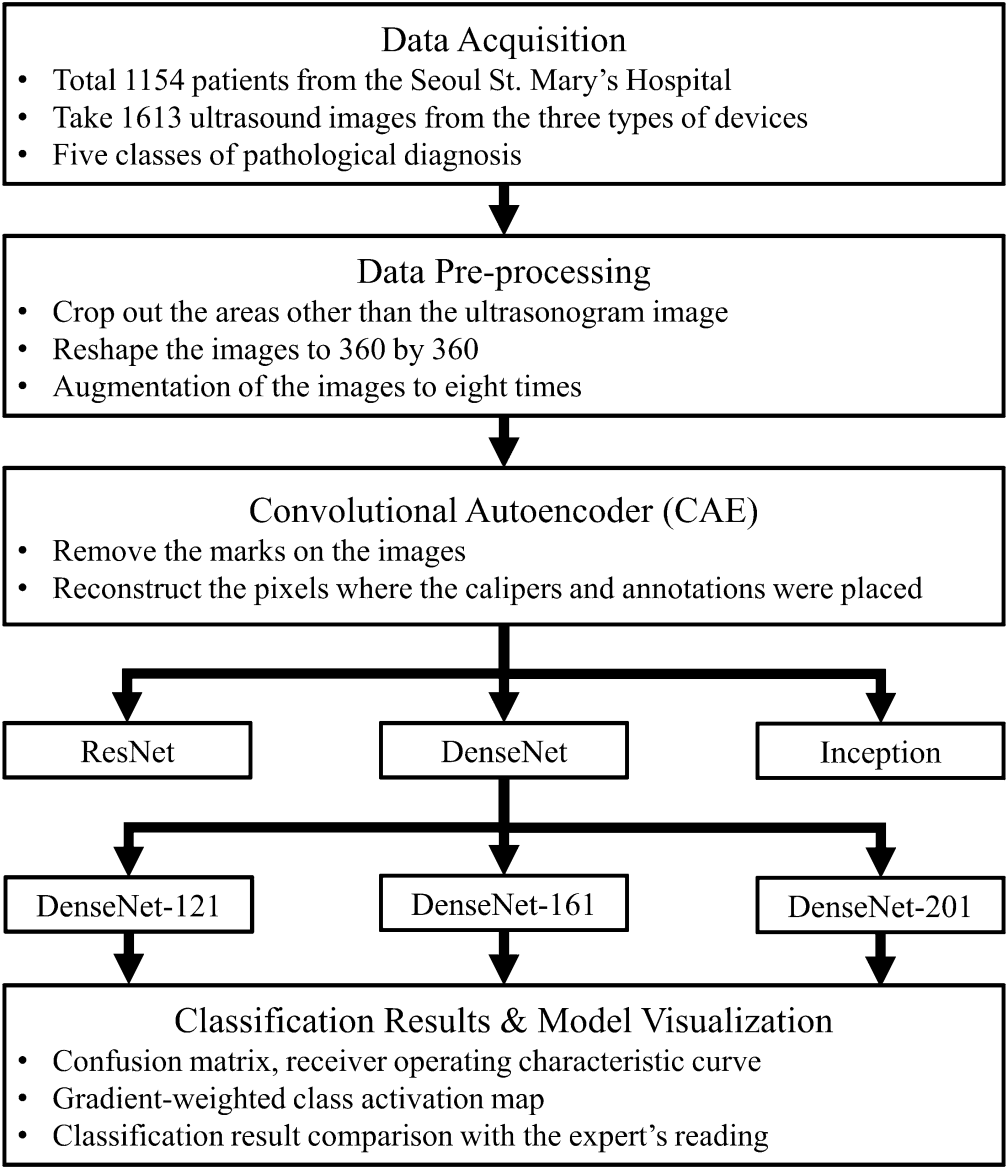
Study flow chart. A deep learning method, a convolutional autoencoder (CAE), was used in the pre-processing stage, and hyper-parameter tuning was conducted in the deep learning model training process.

The research algorithm is shown in Fig. 5. The collected images went through the pre-processing stage to eliminate the effects of different devices and conditions. As the marks on the collected images can affect model training, we removed the marks through the CAE. After the CAE model successfully removed the marks on the images, these processed images were used to train and validate the CNN model. In the final stage, the results were compared against expert readings to validate the results, and CNN visualization methods were used to verify reliability.

### Dataset

This study was conducted using 1613 single-ovary ultrasound images from 1154 patients at the Seoul St. Mary’s Hospital, who underwent surgical removal of tumors of one or both ovaries between January 2010 and March 2020 and had known pathology diagnoses. Preoperative ultrasound images were obtained by using a GE Voluson E8 Expert ultrasound system (GE Healthcare, Milwaukee, WI, USA), an Accuvix XQ (*Medison*, Seoul, Korea), or a WS80A Elite (*Samsung* Medison. Co., Ltd, Seoul, Korea). Representative grayscale images of the tumors were made by the expert ultrasonographer, and all of the images were stored in JPG digitized format. The information from color Doppler was not taken into account in this study. The images were categorized according to the pathology diagnosis as (1) normal (no lesion), (2) cystadenoma (mucinous/serous), (3) mature cystic teratoma, (4) endometrioma, and (5) malignancy, including cancerous and borderline tumors. Representative images are shown in Fig. 1.

### Ethical approval

This study was approved by the Institutional Review Board (IRB) of Seoul St. Mary’s Hospital of the Catholic University of Korea College of Medicine (IRB approval no. KC18RESI0792). All procedures performed in studies involving human participants were carried out in accordance with the Helsinki Declaration ethical principles for medical research. The requirement for informed consent was waived because of the retrospective study design after in-depth review by IRB.

We separated the 1613 images into five data subsets for 5-fold cross-validation. The five subsets were used for training and validation iteratively, yielding robust results from each independent result. Since only one ultrasound image was taken for each ovary, there were no instances of duplication of images of the same ovary in the training and validation data. The data overview is shown in Table 3. All images were processed by the following steps: (1) the frame information for the ultrasound images (patient identification number, name, and timestamp) was removed; (2) the ultrasound images were trimmed to a 1:1 ratio, as not to distort the scale and shape of the original image; and (3) in the final step, the pixel values of the ultrasound images pixel were normalized to (0, 1), and the image resolution was resized to 360 × 360 considering the compatibility between CAE and CNN model. For model training, we performed offline data augmentation only on the training images to 8× by rotating the images by 90° 3 times and flipping each rotated image horizontally.

**Table 3:** The number of patients and images used in the fivefold cross-validation of the classification model.

Types	Normal	Cystadenoma	Mature cystic teratoma	Endometrioma	Malignancy	Total
Patients	145	245	324	196	244	1,154
Image	214	272	364	224	539	1,613
Fold-1	171/43	217/55	291/73	179/45	431/108	1,289/324
Fold-2	171/43	217/55	291/73	179/45	431/108	1,289/324
Fold-3	171 /43	218/54	291/73	179/45	431/108	1,290/323
Fold-4	171/43	218/54	291/73	179/45	431/108	1,290/323
Fold-5	172/42	218/54	292/72	180/44	432/107	1,294/319

### Methods

**Figure 6:**
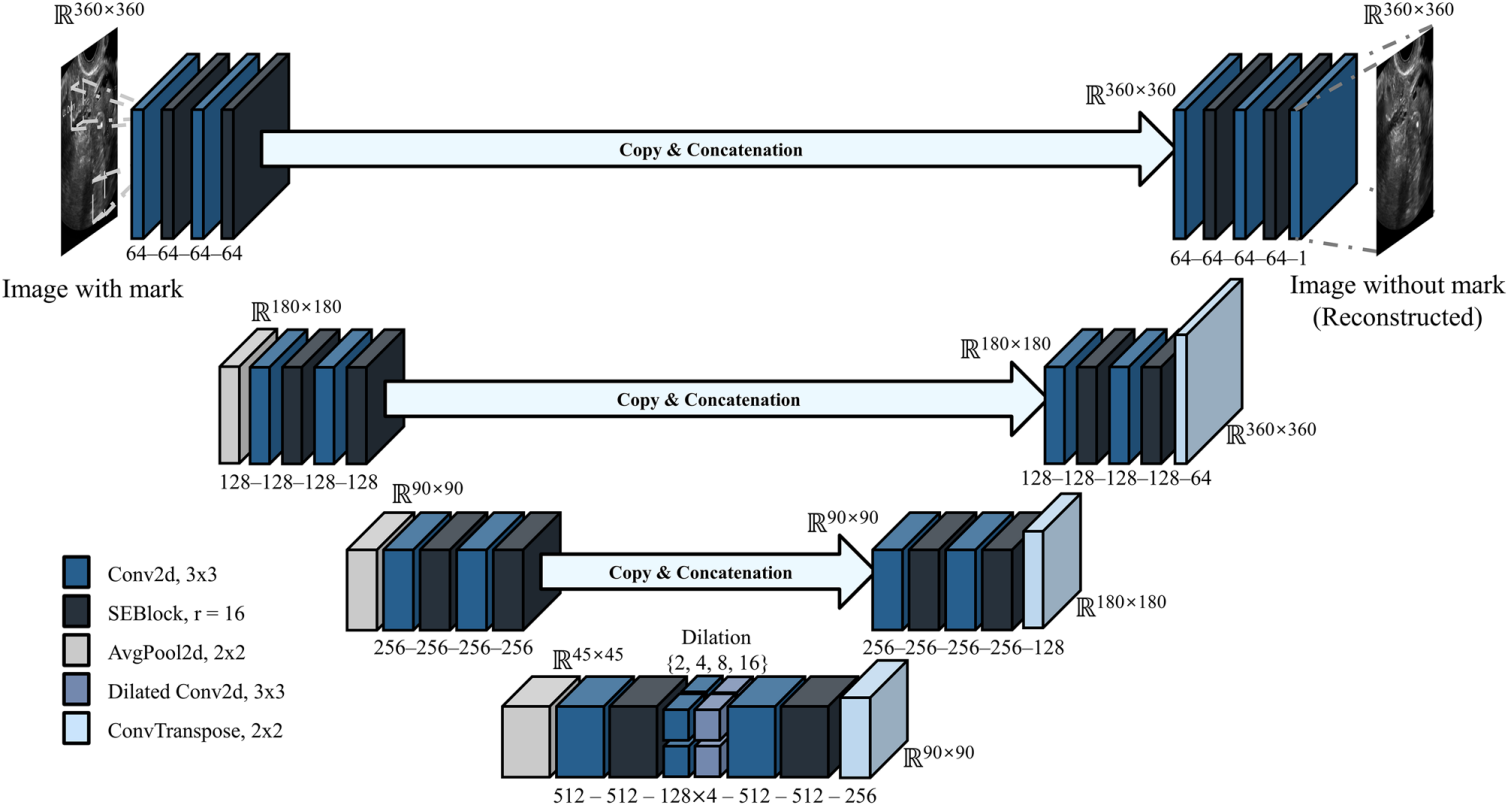
The architecture of the convolutional autoencoder model. This model is designed to remove marks on images and generate high-resolution pixels to replace the marks.

The images processed by the data acquisition methods described above still contained marks, such as calipers and annotations, which cannot be removed manually. These marks can affect the features for classifying each class. Therefore, we designed a CAE model that removed the marks on images and regenerates the pixels where the marks were placed. The structure of the CAE model is shown in Fig. 6. The U-net structure was referenced and minor variations, such as squeeze and excitation block (SE-block) and multi-kernel dilated convolution, were adapted to improve the mark removal performance^13^.

The operation of the CAE model is performed in a series of steps. First, the original image with the marks is input and passed through the squeezing and excitation block, which compresses and weights the information and features of the convolution layer^14^. These feature maps pass into deeper layers by average pooling. In the deepest layer, feature maps are merged with context information via dilated convolution operations with multiple dilation values. After passing through convolution and transposed convolution layers, the feature maps are generated to the clean ultrasound image. During this process, the high-resolution information of the shallow layer is concatenated to the convolution layers to enhance resolution. The CAE model was trained on 171 pairs of marked and clean ultrasound images by setting the following training parameters: 200 epochs, 2 batch size, mean squared loss, Adam optimizer, and 0.00005 learning rate decaying every epoch for 0.95 times. These 171 pairs of marked and clean ultrasound images were collected by saving images separately before adding calipers and annotations to ultrasound images. The marked ultrasound image was input to the CAE model, and the image result of the CAE model was compared to the clean image. The weight parameters of the CAE model are optimized to minimize a mean squared error between two images.

**Figure 7:**
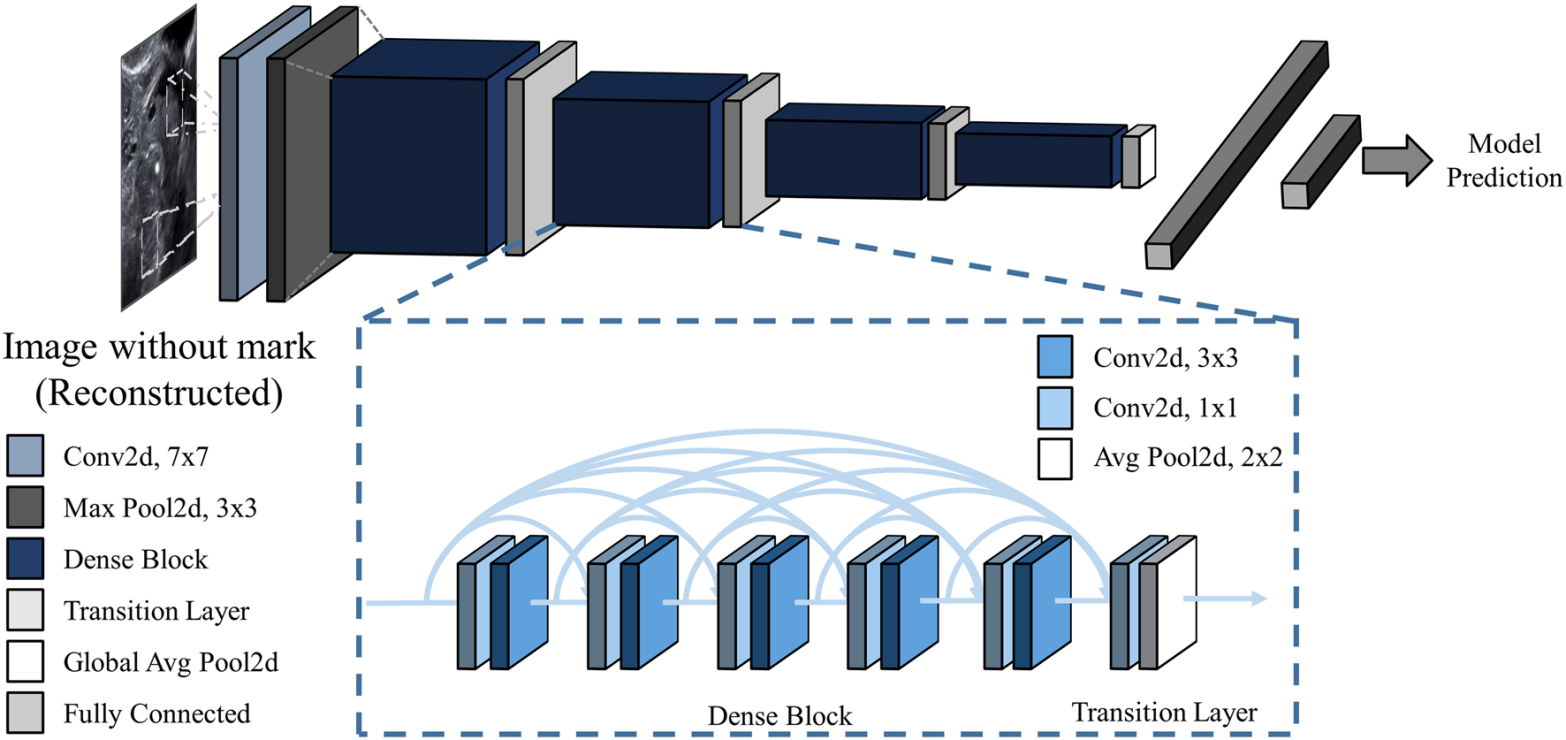
Structure of the DenseNet model. DenseNet uses a DenseBlock that employs fewer parameters while enhancing the information flow and gradient flow.

The purpose of this study was to produce a deep learning model for classifying ovarian tumors from ultrasound images. To date, deep learning models that have demonstrated success in the field of image recognition have mostly been valid in general RGB images. Unlike RGB images, which directly capture reflected light, ultrasound images are obtained indirectly from sound waves reflected from the layers between different tissues. As a result, ultrasound images have low resolution and hazy object boundaries. Considering these differences between ultrasound images and RGB images, it is as yet unknown which deep learning model is optimal for classifying ultrasound images. We trained three CNN models ResNet^15^, inception-v3^16^, and DenseNet^17^ architecture, that have been widely used and validated in many other classification tasks, to discover the best model for classification of ovarian tumors on ultrasound images. Each model's end layer was modified to have five hidden nodes to classify five classes of ovarian ultrasound images. The model weights, except for the end layer, were initialized with pre-trained parameters that were optimized for another computer vision task (ImageNet). We newly trained these models, ResNet101, Inception48, and DenseNet121, for the task of distinguishing the five classes by setting the following training parameters: 50 epochs, 8 batch size, cross-entropy loss, Adam optimizer, and 0.00005 learning rate decaying every epoch for 0.95 times. Among the models, the DenseNet121 structure showed best result on the validation dataset, and we additionally trained other DenseNet models having a different number of convolutional layers. The results for each structure are shown in Table 4 and Fig. 7, respectively.

**Table 4:** Five-fold cross-validation of the accuracy of the convolutional neural network models with 95% confidence interval (in parentheses) calculated on the fivefold validation dataset using Student’s *t*-test.

Model type	Layers (n)	Accuracy (%)
ResNet	101	73.95 (72.75–75.15)
Inception	48	75.00 (73.88–76.12)
DenseNet	121	82.96 (81.12–84.81)
	161	83.27 (81.35–85.2)
	201	79.51 (77.78–81.23)
DenseNet without CAE	161	74.20 (72.35–76.05)

### Evaluation measures

For the evaluation of the ovarian tumor classification, sensitivity, specificity, positive predictive value (PPV), negative predictive value (NPV), accuracy, and area under the receiver operating characteristic (ROC) curve (AUC) were used as performance measures. The mean and 95% confidence interval of the five-fold cross-validation results were used to calculate performance measures.

## Data Availability

The datasets used and/or analysed during the current study available from the corresponding author on reasonable request.
