# Author Correction: Ovarian tumor diagnosis using deep convolutional neural networks and a denoising convolutional autoencoder

**DOI:** 10.1038/s41598-023-28524-0

**Published:** 2023-01-25

**Authors:** Yuyeon Jung, Taewan Kim, Mi-Ryung Han, Sejin Kim, Geunyoung Kim, Seungchul Lee, Youn Jin Choi

**Affiliations:** 1 Department of Obstetrics and Gynecology, Soonchunhyang University Bucheon Hospital, Soonchunhyang University College of Medicine, Bucheon, Republic of Korea; 2 Department of Mechanical Engineering, Pohang University of Science and Technology (POSTECH), Pohang, Republic of Korea; 3 Division of Life Sciences, College of Life Sciences and Bioengineering, Incheon National University, Incheon, Republic of Korea; 4 Department of Obstetrics and Gynecology, Seoul St. Mary’s Hospital, College of Medicine, The Catholic University of Korea, Seoul, Republic of Korea; 5 Graduate School of Artificial Intelligence, Pohang University of Science and Technology (POSTECH), Pohang, Republic of Korea; 6 Institute of Convergence Research and Education in Advanced Technology, Yonsei University, Seoul, Republic of Korea; 7 Cancer Research Institute, College of Medicine, The Catholic University of Korea, Seoul, Republic of Korea

Correction to: *Scientific Reports* https://doi.org/10.1038/s41598-022-20653-2, published online 11 October 2022

The original version of this Article contained an error in the Author Information section.

“These authors contributed equally: Yuyeon Jung, Taewan Kim, Seungchul Lee and Youn Jin Choi.”

now reads:

“These authors contributed equally: Yuyeon Jung and Taewan Kim. These authors jointly supervised this work: Seungchul Lee and Youn Jin Choi.”

The original Article has been corrected.

